# P-1290. Invasive group A streptococcal infection in children in Toronto, Canada

**DOI:** 10.1093/ofid/ofae631.1471

**Published:** 2025-01-29

**Authors:** Halima Dabaja Younis, Jennie Johnstone, Zoe Zhong, Caroline Kassee, Vanessa Allen, Irene Armstrong, Mahin Baqi, Kevin R Barker, Ari Bitnun, Sergio Borgia, Aaron Campigotto, Sumon Chakrabarti, Wayne Gold, Alyssa Golden, Christopher Kandel, Ian Kitai, Julianne Kus, Liane Macdonald, Irene Martin, Matthew Muller, Jeya Nadarajah, Daniel R Ricciuto, David Richardson, Medina Saffie, Manal Tadros, Monali Varia, Kazi Hassan, Maxime Lefebvre, Angel Li, Asfia Sultana, Gloria Crowl, Lubna Farooqi, Mare Pejkovska, Nadia Malik, Shiva Barati, Tamara Vikulova, Allison McGeer

**Affiliations:** Sinai Health, Toronto, Ontario, Canada; University of Toronto, Toronto, Ontario, Canada; Sinai Health System, University of Toronto, Toronto, Ontario, Canada; Sinai Health, Toronto, Ontario, Canada; Sinai Health System, University of Toronto, Toronto, Ontario, Canada; Toronto Public Health, Toronto, Ontario, Canada; William Osler Health System, Toront, Ontario, Canada; Trillium Health Partners, Toronto, Ontario, Canada; Hospital for Sick Children, Toronto, ON, Canada; William Osler Health System, Toront, Ontario, Canada; Hospital for Sick Children, Toronto, ON, Canada; Trillium Health Partners, Toronto, Ontario, Canada; University of Toronto, Toronto, Ontario, Canada; University of Manitoba, Winnipeg, Manitoba, Canada; Michael Garron Hospital, Toronto, Ontario, Canada; University of Toronto, Toronto, Ontario, Canada; Public Health Ontario, Toronto, Ontario, Canada; Public Health Ontario, Toronto, Ontario, Canada; National Microbiology Laboratory (NML), Winnipeg, MB, Canada; Unity Health, University of Toronto, Toronto, Ontario, Canada; Oak Valley Heatlh, Toronto, Ontario, Canada; Lakeridge Health, Oshawa, Ontario, Canada; William Osler Health System, Toront, Ontario, Canada; Joseph Brant Hospital, Burlington, Ontario, Canada; The Hospital for Sick Children, Toronto, Ontario, Canada; Peel Public Health, Mississauga, Ontario, Canada; Sinai Health System, Toronto, Ontario, Canada; Sinai Health, Toronto, Ontario, Canada; Sinai Health System, Toronto, Ontario, Canada; Sinai Health, Toronto, Ontario, Canada; Sinai Health, Toronto, Ontario, Canada; Sinai Health System, University of Toronto, Toronto, Ontario, Canada; Sinai Health System, Toronto, Ontario, Canada; Sinai Health, Toronto, Ontario, Canada; Sinai Health, Toronto, Ontario, Canada; Sinai Health, Toronto, Ontario, Canada; Mt. Sinai Hospital, Toronto, Ontario, Canada

## Abstract

**Background:**

Rising cases of invasive group A streptococcus (iGAS) in children have been reported globally. We assessed the epidemiology of pediatric iGAS over three decades.

**Figure:**
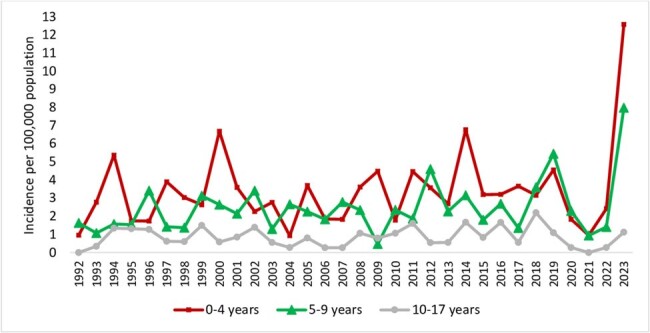

The annual incidence of invasive GAS infections in children in Toronto and Peel Region during the years 1992-2023

**Methods:**

Since 1992, the Toronto Invasive Bacterial Diseases Network has conducted surveillance for iGAS in Toronto/Peel Region, Canada. The National Microbiology Laboratory provides *emm* typing, and Statistics Canada supplies population data. iGAS data were analyzed by age group—toddlers (0-4 yrs), young children (5-9 yrs), and older children (10-17 yrs)— and over time from 1992 to 2024.

**Figure:**
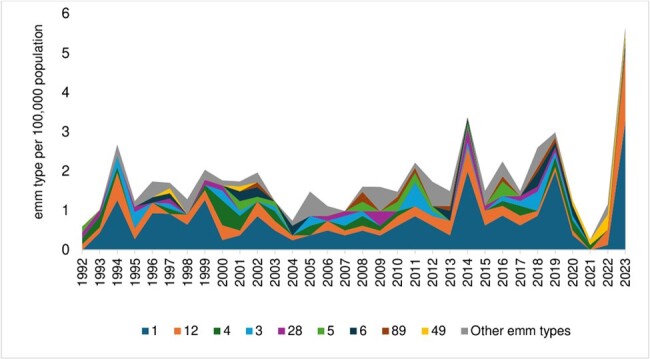

emm types in pediatric cases per 100,000 population in Toronto and Peel Region from 1992 to 2023.

**Results:**

506 pediatric iGAS cases were identified. Figure 1 shows the incidence by age group. 61% of cases were male; 33% had underlying conditions. Main presentations were soft tissue infection (30%), bacteremia with no focus (22%), bone/joint infections (20%) and pneumonia (16%). Streptococcal toxic shock syndrome (STSS) occurred in 5.7% of cases, and necrotizing fasciitis (NF) in 2.6%. Positive blood cultures were found in 71% of cases, with additional sterile site cultures in 20%. *emm1* (42%) and *emm12* (16%) were the most common types (Figure 2), with M1_UK_ comprising 63% of *emm1* isolates in 2022/2023.

During the period of pandemic restrictions (Mar 2020-Apr 2022), iGAS cases declined, and *emm1* disappeared. As restrictions eased, *emm1* and, to a lesser extent, *emm12* rebounded (Figure 3). Seasonality, with increased winter/spring cases, became more pronounced (Figure 4).

Toddlers had higher rates of bacteremia without focus (27% vs. 14%, p=0.01) and *emm12* (20% vs. 8.4%, p=0.02) than older children. Younger children had higher rates of bone/joint infections than toddlers (28% vs. 16%, p=0.004).

Rates of STSS, NF, and 30-day mortality were consistent across age groups and time, with no association to specific *emm* types, except for higher mortality in *emm4* [12.5% vs. 3.6%, OR 3.8 (1.2-12.1)]. Increasing pneumonia rates over time were associated with *emm1*. In multivariate analysis, pneumonia [OR 7.2 (2.9-17.6)], NF [OR 15.5 (4.2-58)], and increased age [OR 1.8 (1.03-3.2)] predicted STSS.

**Figure:**
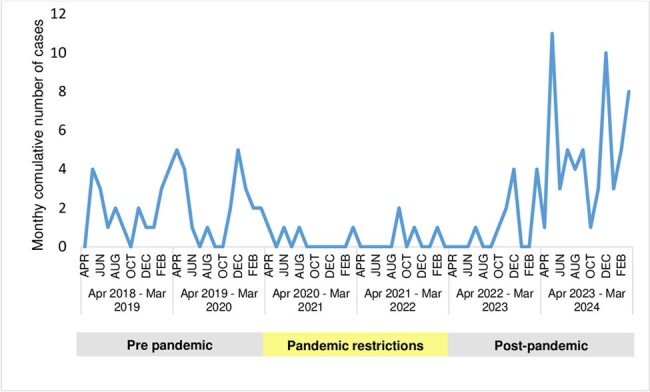

Monthly pediatric iGAS cases in relation to pandemic public health restrictions in Toronto and Peel Region from April 2018 to March 2024.

**Conclusion:**

The recent rise in pediatric iGAS infections is a public health concern. Incidence has increased, but severity remains unchanged. *emm1* transmission seems more susceptible to control by public health measures. Continuous surveillance is essential.

**Figure:**
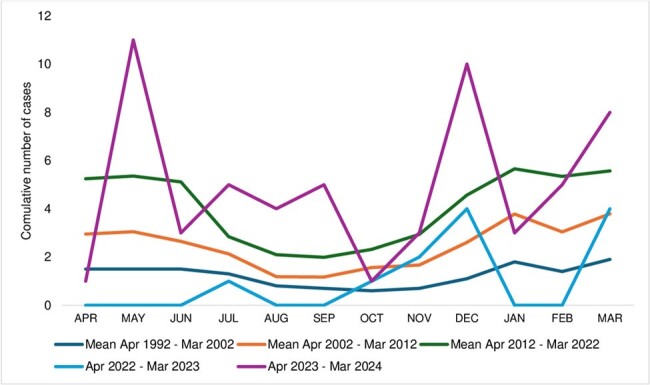

Seasonality of iGAS in children in Toronto/Peel region, 1992-2023

**Disclosures:**

**Allison McGeer, MD**, AstraZeneca: Honoraria|GSK: Honoraria|Merck: Honoraria|Moderna: Honoraria|Novavax: Honoraria|Pfizer: Grant/Research Support|Pfizer: Honoraria|Roche: Honoraria|Seqirus: Grant/Research Support|Seqirus: Honoraria

